# Impacts of intentional mycoplasma contamination on CHO cell bioreactor cultures

**DOI:** 10.1002/bit.27161

**Published:** 2019-09-11

**Authors:** Erica J. Fratz‐Berilla, Talia Faison, Casey L. Kohnhorst, Sai Rashmika Velugula‐Yellela, David N. Powers, Kurt Brorson, Cyrus Agarabi

**Affiliations:** ^1^ U.S. Food and Drug Administration, Center for Drug Evaluation and Research, Office of Product Quality, Office of Biotechnology Products, Division of Biotechnology Review and Research II Silver Spring Maryland; ^2^ Currently with Nova Biomedical Waltham Massachusetts; ^3^ Currently with Parexel International Corporation Waltham Massachusetts

**Keywords:** Chinese hamster ovary (CHO) cell culture, monoclonal antibody (mAb), mycoplasma

## Abstract

Mycoplasma contamination events in biomanufacturing facilities can result in loss of production and costly cleanups. Mycoplasma may survive in mammalian cell cultures with only subtle changes to the culture and may penetrate the 0.2 µm filters often used in the primary clarification of harvested cell culture fluid. Culture cell‐based and indicator cell‐based assays that are used to detect mycoplasma are highly sensitive but can take up to 28 days to complete and cannot be used for real‐time decision making during the biomanufacturing process. To support real‐time measurements of mycoplasma contamination, there is a push to explore nucleic acid testing. However, cell‐based methods measure growth or colony forming units and nucleic acid testing measures genome copy number; this has led to ambiguity regarding how to compare the sensitivity of the methods. In addition, the high risk of conducting experiments wherein one deliberately spikes mycoplasma into bioreactors has dissuaded commercial groups from performing studies to explore the multiple variables associated with the upstream effects of a mycoplasma contamination in a manufacturing setting. Here we studied the ability of *Mycoplasma arginini* to persist in a single‐use, perfusion rocking bioreactor system containing a Chinese hamster ovary (CHO) DG44 cell line expressing a model monoclonal immunoglobulin G1 (IgG1) antibody. We examined *M. arginini* growth and detection by culture methods, as well as the effects of *M. arginini* on mammalian cell health, metabolism, and productivity. We compared process parameters and controls normally measured in bioreactors including dissolved oxygen, gas mix, and base addition to maintain pH, to examine parameter changes as potential indicators of contamination. Our work showed that *M. arginini* affects CHO cell growth profile, viability, nutrient consumption, oxygen use, and waste production at varying timepoints after *M. arginini* introduction to the culture. Importantly, how the *M. arginini* contamination impacts the CHO cells is influenced by the concentration of CHO cells and rate of perfusion at the time of *M. arginini* spike. Careful evaluation of dissolved oxygen, pH control parameters, ammonia, and arginine over time may be used to indicate mycoplasma contamination in CHO cell cultures in a bioreactor before a read‐out from a traditional method.

## Graphical abstract

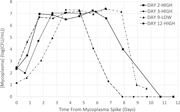

## 1 INTRODUCTION

Mollicutes, commonly referred to as Mycoplasma, are a genus of bacteria that are challenging to detect and remove from mammalian cell culture. Mycoplasma are the smallest, mostly self‐replicating bacteria (0.1–0.3 µm in diameter); the organism's pleomorphic nature due to the lack of a cell wall allows these bacteria to penetrate the typical 0.1–0.22 µm sterilizing‐grade filters used in biomanufacturing. Mycoplasma can potentially contaminate a bioprocessing scheme through raw materials, such as cell culture media components (Drexler & Uphoff, 2002; Kljavin, 2011), and during steps that require manual manipulation of cell lines such as cell banking and cell line development (Nikfarjam & Farzaneh, 2012). Mycoplasma have small genomes which limits metabolic and replication options and typically require a host cell for survival. Some species of mycoplasma have caused occult contaminations of cell cultures, especially in basic research settings, with minimal visible changes to cell health or cell culture performance. However, mycoplasma can alter the culture performance and product quality in more subtle ways through several potential mechanisms including competition for culture nutrients, induction of abnormal cell growth and cytopathic effects by invading or fusing with the host cells, and alteration of the host cell expression profile (Rottem, 2003).

The ability of mycoplasma to evade detection and grow in mammalian cell cultures creates risk to patients that receive injectable biopharmaceuticals. Yet, there is sparse literature on the details of kinetics and process impacts by mycoplasma contamination in commercial upstream biomanufacturing. Usually when firms find mycoplasma in their cultures, they immediately decontaminate after taking a limited number of culture samples for specification and test raw material samples to trace the source of the contamination. Some published studies regarding mycoplasma species contaminating mammalian cell lines have been conducted in cell culture flasks, shake flasks, spinner flasks, and other standard cell culture vessels (Dabrazhynetskaya et al., 2011; David, Volokhov, Ye, & Chizhikov, 2010; Faison et al., 2018; Laborde et al., 2010; Wang et al., 2017), but not bioreactors; GMPs preclude introducing mycoplasma to a cell culture facility. However, unlike culture vessels, bioreactors can both monitor and control process parameters as well as perfuse fresh media and remove waste materials at a controlled rate. Thus, bioreactors are a much better process model for commercial upstream operations than shake flasks. Further, many of the above‐described studies were performed using serum‐containing media, which is also not realistic in a commercial setting. While it has been shown that mycoplasma are killed and cleared in a typical downstream bioprocessing scheme (Wang et al., 2017) which reduces the risk for contamination carryover to drug substance, the risk and impact of mycoplasma contamination in relevant models of upstream bioprocessing is less understood in terms of culture performance and product quality effects.

Conventional assays commonly employed for mycoplasma testing take 14–28 days to complete and require labor‐intensive manipulation (FDA, 1993, 2010). To facilitate more rapid detection, some biomanufacturers have begun to adopt nucleic acid test (NAT) methods as well as other rapid microbial techniques. Thus far, challenges in demonstrating comparability and method validation have hampered adoption. Here we developed models of early‐stage process and late‐stage process bioreactor culture contamination events using *M. arginini* and Chinese hamster ovary (CHO) cells grown in serum‐free medium expressing a model immunoglobulin 1 (IgG1) product to examine the growth kinetics of *M. arginini* in a controlled bioreactor environment and the effects on CHO cell culture performance and process parameters. Our data support the understanding of what a mycoplasma contamination event may look like in a typical biomanufacturing scheme and provide knowledge of how process monitoring may identify a mycoplasma contamination event in the manufacturing environment. Additionally, this study provides data for the feasibility and benefits for development and implementation of rapid mycoplasma testing, such as NAT methods, in upstream biomanufacturing.

## 2 MATERIALS AND METHODS

### 2.1 Seed train expansion and inoculum preparation

This experiment used a previously described recombinant CHO DG44 cell line that expresses a model chimeric IgG1 (Velugula‐Yellela, Williams, et al., 2018). Frozen cell stocks (2 × 10^7^ cells/ml) were thawed and seeded into multiple 1 L spinner flasks containing 300 ml CD OptiCHO (Life Technologies; A11222) media supplemented with 8 mM l‐glutamine (Corning; 25‐005‐CV). Spinner flasks were incubated at 37°C and 8% CO_2_ at an agitation speed of 73 rpm. Fresh media was added to double the volume after the viable cells reached ≥2 × 10^6^ cells/ml. The next day (day before inoculation), the total volume of each spinner flask was brought up to 1 L using fresh media. On the day of inoculation, cells were pelleted by centrifugation at 300*g* for 10 min at 23°C and resuspended in fresh CD OptiCHO media supplemented with 8 mM l‐glutamine and 1× Soy Hydrolysate (Sigma‐Aldrich; 58903C or S1674). Soy hydrolysate was added to the media to support growth of *M. arginini* in serum‐free media based on previous studies (Wang et al., 2017). Inoculum cell density was measured, and each bioreactor was inoculated with identical volumes to reach the target seeding density of 1 × 10^6^ cells/ml.

### 2.2 Rocking bioreactor operation

A GE ReadyToProcess WAVE™ 25 rocker was operated in dual mode with two 2 L single‐use bioreactors (1 L maximum operating volume) containing porous polyethylene‐based perfusion filters (CB0002L10‐34). Three 14–19 day runs of two bioreactors (*n* = 6) were completed with identical setpoints and strategies as listed in Table 1. Six bioreactors were run in total, but for the third run Day 12‐High was treated as a control for Day 9‐Low until Day 12, and from Day 12 onward was used for a late‐stage contamination event bioreactor and served as an internal control to itself (Table 2). With the exceptions of rocking speed and rocking angle, culture parameters were automatically controlled using the UNICORN system control software. Bioreactors were run in batch mode until glutamine reached ≤ 1 mM, except for Day 2‐High and Day 2‐Control in which a glutamine feed of 2 mM was added 1 day before the start of perfusion. Cell bleeds were generally performed when cells measured above 55 × 10^6^ cells/ml to bring cell density to 20–40 × 10^6^ cells/ml, depending on the batch age and desired cell density reduction.

**Table 1:** Culture parameters for 1 L perfusion bioreactor cultures

Culture parameter	Setpoint or description
Bioreactor volume	2 L
Culture volume	1 L
Gas mix flow rate	0.3 L/min, fixed flow
O_2_ mix range	21–50%
CO_2_ mix range	0–15%
Temperature	37°C
pH	7.1
pH control method	CO_2_/base (0.5 M NaOH)
Dissolved oxygen	50% air saturation
Dissolved oxygen control method	O_2_ (rocking speed/angle control manually)
Rocking speed	20–28 rocks per minute (rpm)
Rocking angle	6–10°
Perfusion start criteria	Glutamine≤1 mM
Perfusion rate	1–3.5 L/day
Cell bleed criteria	VCD≥55 × 10^6^ cells/ml
Harvest criteria	CHO cell viability of spiked bioreactor <50%

**Table 2:** Description of bioreactor runs

Bioreactor name	Run length (Days)	Day of mycoplasma spike	Mycoplasma spike level (CFU/ml)	Description
Day 2‐High	14	2	220	Model for early‐stage high‐level bioreactor contamination
Day 2‐Control	14	–	–	Control bioreactor (uncontaminated)
Day 3‐High	14	3	260	Model for early‐stage high‐level bioreactor contamination
Day 3‐Control	14	–	–	Control bioreactor (uncontaminated)
Day 9‐Low	19	9	15	Model for late‐stage low‐level bioreactor contamination
Day 12‐High	19	12	300	Model for late‐stage high‐level bioreactor contamination and control for Day 9‐Low bioreactor up to Day 12

### 2.3 Mycoplasma preparation and spiking

The mycoplasma spike for the bioreactor was prepared from a frozen stock of *M. arginini* in 45% glycerol thawed at room temperature in a laminar flow hood using a sterile syringe and disposable pipette basin. The mycoplasma stock was mixed with 3 ml media (OptiCHO/l‐Glutamine + soy hydrolysate) warmed to 37°C to seed a final target concentration of 10^1^ (low spike) or 10^2^–10^3^ (high spike) CFU/ml in 1 L bioreactor volume. A negative control containing an identical volume of media and supplements only was also prepared for the uncontaminated bioreactor. Syringes were attached to the WAVE bioreactor sample ports, the contents were dispensed, and the attached syringes were used to flush the sample line with bioreactor media before disconnecting. The initial mycoplasma titration samples for time 0 were taken approximately 1–2 hr post‐spike to allow for contaminant and clump dispersal. As mycoplasma doubling times are about 6 hr or more, this lag is unlikely to lead to titer overestimates (Waites & Talkington, 2004).

### 2.4 Perfusion of single‐use bioreactors

As previously noted, bioreactors were run in batch mode until glutamine reached ≤ 1 mM, apart from Day 2‐High and Day 2‐Control in which a glutamine feed of 2 mM was added 1 day before the start of perfusion. Perfusion was started for each run at a rate of 1 L/day, based on the results of previous experiments (data not shown). In general, to keep glutamine ≥ 0.5 mM and glucose ≥ 0.5 g/L, perfusion was increased to 2 L/day when cells were > 10 × 10^6^ cells/ml, 3 L/day when cells were > 20 × 10^6^ cells/ mL, and the maximum rate used of 3.5 L/day when cells were measured > 40 × 10^6^ cells/day. Freshly prepared media bottles were stored no more than 7 days at 4°C protected from light and were manually attached each day at roughly the same time and perfusate collection bottles were exchanged out for empty bottles. Raw samples from each day's perfusate were retained for mycoplasma testing and titer measurements. Perfusion of contaminated bags was discontinued when CHO cell viability had dropped below ~30%.

### 2.5 In‐process sampling

Daily morning and evening samples (~8 hr apart) were removed from both cultures and analyzed using the BioProfile FLEX analyzer (Nova Biomedical) to measure viable cell density (VCD), pH, glutamine, glucose, lactate, glutamate, and ammonium. Remaining sample volume was aliquoted and prepared for further analysis and testing. Samples for mycoplasma detection by plating and colony counting were prepared in 45% glycerol suspension, mixed by 2–3 inversions of the tube, and set at room temperature for 10–15 min before placement at −20°C until the day of dilution and plating. Samples for titer measurements were clarified by centrifugation at 300*g* for 5 min at 4°C and sterile filtered using 0.22 µm PVDF filters. Cell‐free samples were frozen and stored at −20°C until future analysis.

### 2.6 Mycoplasma quantification

*Mycoplasma arginini* strain 23243 (ATCC, Manassas, VA) was cultivated with SP4 + arginine medium agar and broth (Hardy Diagnostics). In‐process samples from both control and spiked bioreactors were plated, and colonies were counted (Wang et al., 2017). Briefly, mycoplasma titers in bioreactor test articles were determined by performing serial 10‐fold dilutions using phosphate‐buffered saline (PBS) (Gibco, Carlsbad, CA) and 100 µL of each dilution was plated on SP4 + arginine medium agar. Colonies were counted 5 days after plating and all samples were plated in duplicate.

### 2.7 Amino acid characterization by LC‐MS

Crude bioreactor media was centrifuged and passed through a 0.22 μm filter. A perchloric acid cleanup was used to remove protein and particulate matter, which involved mixing filtered bioreactor media with 0.4 N HClO_4_ at a 1:1 ratio and centrifuging at 1962*g* for 5 min at RT. The clarified media was collected to be analyzed by LC‐MS.

A Waters Xevo G2 Q‐ToF (run in ESI‐positive sensitivity mode) coupled to a Waters ACQUITY UPLC I‐Class was used for analysis. We used an Intrada Amino Acid column (Imtakt) to perform normal phase chromatography and separate the amino acids. The buffers used were A: acetonitrile + 0.1% formic acid and B: 100 mM ammonium formate, with a flow rate of 0.6 ml/min, a gradient time of 15 min, and column temperature of 40°C. Amino Acid Standards (Agilent) were utilized to generate a calibration curve 20–2700 pmol/μL) in the QuanLynx software (Waters), which was used to calculate the concentrations of amino acids detected in the prepared bioreactor media samples. Media samples were run in triplicate, with error bars indicating standard deviations. Additional information on this method can be found in past work (Velugula‐Yellela, Kohnhorst, et al., 2018).

### 2.8 Titer measurements using protein A bio‐layer interferometry (BLI) sensors

Samples were thawed at room temperature and titer was measured using Protein A dip‐and‐read BLI sensors (FortéBio, 18‐5010) on the Octet RED96 system (FortéBio) as described previously (Velugula‐Yellela, Kohnhorst, et al., 2018). Cell‐specific IgG1 production rates (*Q*_p,_ pg·cell^−1^·day^−1^) for each perfusion day were calculated using the following adapted formula (Clarke et al., 2011)

$$Q_{p}(\text{pg}\;\cdot \;{\text{cell}}^{-1}\;\cdot \;{\text{day}}^{-1})=\left[\frac{T_{f}-T_{i}}{C_{f}-C_{i}}\right]\times \text{daily}\;\;\text{growth}\;\text{rate},$$

where

$$\text{daily}\;\text{growth}\;\;\text{rate}=\frac{\ln\left(C_{f}/C_{i}\right)}{t_{f}-t_{i}},$$

*t*_f_ represents the total IgG in the bioreactor at time *t*_f_, collected perfusate between time *t*_f_ and time *t*_i_ (if applicable), and cell bleed that occurred between *t*_f_ and *t*_i_ (if applicable), and *t*_i_ is the IgG in the bioreactor at time *t*_i_; *C*_f_ and *C*_i_ represent the final and initial number of viable cells for a given perfusion day.

## 3 RESULTS AND DISCUSSION

### 3.1 *M. arginini* growth in bioreactors culturing CHO cells

**Figure 1:**
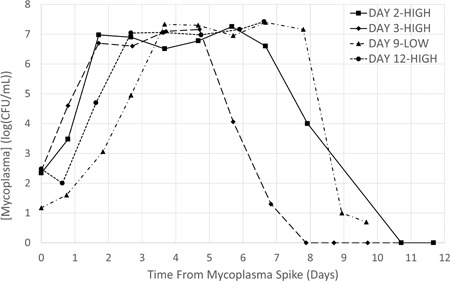
*Mycoplasma arginini* growth profiles in four CHO cell bioreactor cocultures. Bioreactors Day 2‐High and Day 3‐High were spiked with *M. arginini* when CHO VCD reached 2 × 10^6^ cells/ml before the perfusion start day (early‐stage contamination events). *M. arginini* was spiked into Day 9‐Low when CHO VCD reached 10 × 10^6^ cells/ml and in Day 12‐High after a third cell bleed to bring the CHO VCD to 10–15 × 10^6^ cells/ml (late‐stage contamination events). CHO, Chinese hamster ovary; VCD, viable cell density

Two separate early‐stage contamination models (Day 2‐High and Day 3‐High), were conducted with uncontaminated controls (Day 2‐Control and Day 3‐Control). The CHO cells were grown in bioreactors to a density of ≥2 × 10^6^ cells/ml with a viability ≥92% and then were inoculated with 220 and 260 CFU/ml of *M. arginini*, respectively, before the start of perfusion. *M. arginini* appeared to grow exponentially for 2 days, plateauing at a peak density of ~10^7^ CFU/ml for 3–5 days and then decrease exponentially for 2–3 days. After 3 days of decline, no live mycoplasma could be detected (Figure 1) by plate counting. These growth trends mirror those of *M. arginini* in shake flasks under similar culturing conditions, but peak density only reaches approximately 10^6^ CFU/ml (Faison et al., 2018).

Two late‐stage contamination models were investigated with low and high *M. arginini* additions, Day 9‐Low and Day 12‐High, inoculated with 15 and 300 CFU/ml of *M. arginini*, respectively. Both cultures were grown to CHO cell densities of 9–12 × 10^6^ cells/ml with a viability ≥90%, and perfusion was operating at 2 L/day before spiking with *M. arginini*. In the case of Day 12‐High, data were collected as control culture up to Day 12. The bioreactor was then switched over to an experimental culture by bleeding to reach a target VCD of 10–15 × 10^6^ cells/ml before an *M. arginini* spike to match spiking conditions to those of Day 9‐Low. For Day 9‐Low, *M. arginini* grew for 3 days, plateaued at a peak density of ~10^7^ CFU/ml which held steady for 5 days, and then plummeted in 1 day from >10^7^ to 10^1^ CFU/ml (Figure 1). However, live *M. arginini* residuals persisted nearly 10 days after the spike. Interestingly, the low concentration spike (15 CFU/ml) showed a rapid increase in the growth phase to reach the same peak density (10^7^ CFU/ml) as the higher density spike culture (300 CFU/ml). Based on these kinetics, we conclude that the inoculum density of *M. arginini* contamination does not make a significant difference in mycoplasma growth kinetics in the bioreactor environment, rather the overall growth is probably limited by nutritional components in the media. In Day 12‐High, *M. arginini* population decreased slightly during lag phase (first day of inoculum into the CHO culture), possibly due to washout from perfusion at the time of the spike. It then grew exponentially for 2 days, followed by a plateau phase until the run was ended (Figure 1). Overall, *M. arginini* exhibits similar growth kinetics when cocultured with CHO cells in a rocking bioreactor, independent of its inoculum density at the initiation of the model contamination event or the state of the CHO cell growth at the time of contamination.

### 3.2 CHO cell growth and metabolism after contamination with *M. arginini*

**Figure 2:**
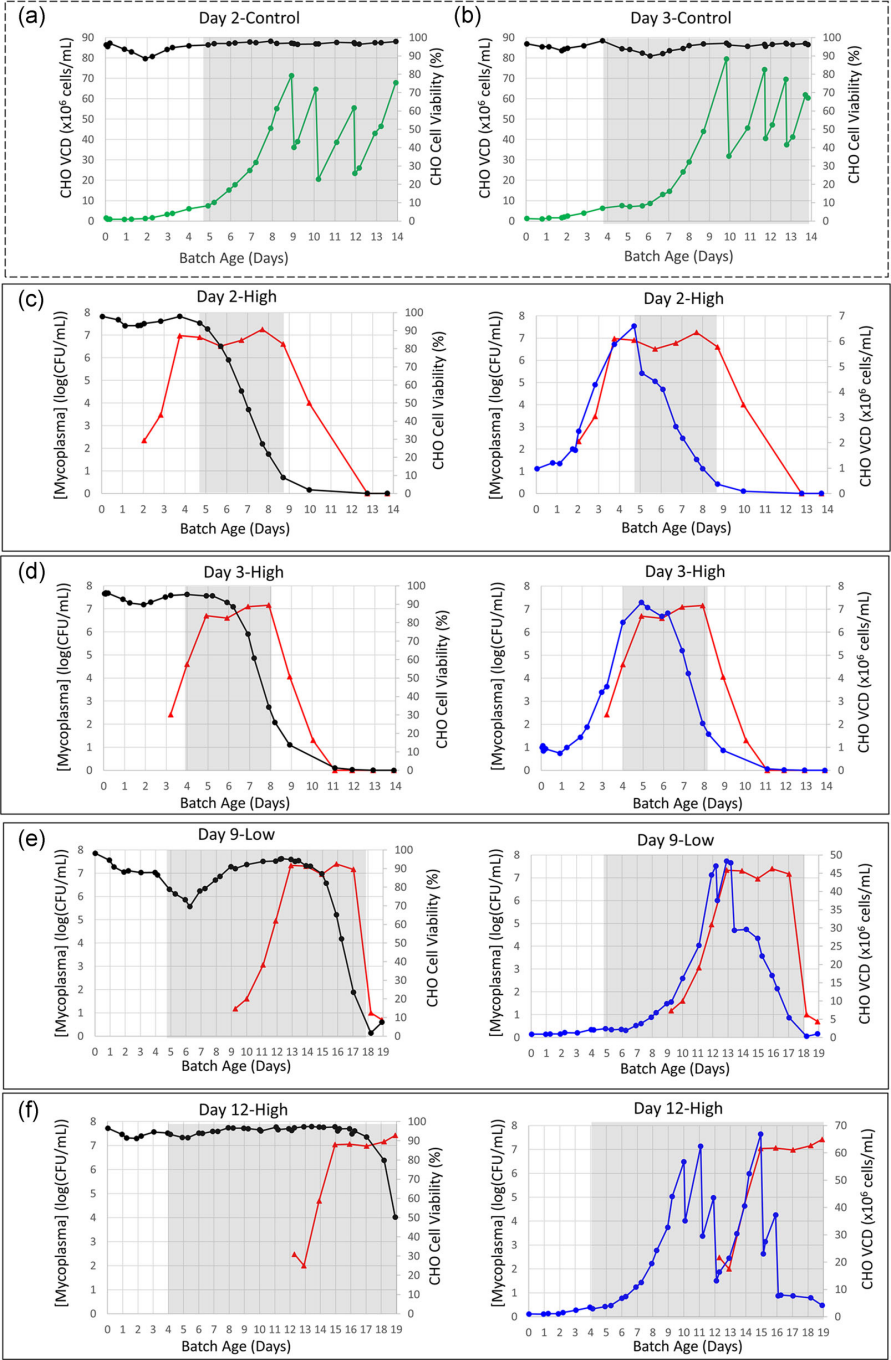
CHO cell growth profiles with and without *Mycoplasma arginini* contamination. (a) Day 2‐Control. (b) Day 3‐Control. (c) Day 2‐High. (d) Day 3‐High. (e) Day 9‐Low. (f) Day 12‐High. In uncontaminated (control) bioreactors within the dashed line box CHO cell growth curves (green) and CHO cell viability (purple) are shown. In contaminated bioreactor models, viable mycoplasma measurements are shown in red, CHO cell viability data are in black, and CHO VCD data are in blue. Grey background signifies the time during which the bioreactor was run in perfusion mode. Steep drops in VCD correspond to manual cell bleeds. CHO, Chinese hamster ovary; VCD, viable cell density [Color figure can be viewed at wileyonlinelibrary.com]

Based on preliminary experiments performed in shake flasks, we found that *M. arginini* coculture resulted in a decrease in CHO cell growth rate and a reduced peak viable cell density (data not shown). However, a more accurate understanding of growth kinetics changes and CHO cell metabolism needed to rule out confounding factors by studying the CHO cells in a more controlled environment. For example, in a controlled bioreactor, the CHO cells could proliferate for a long enough period to capture the entire growth curve for subsequent comparison to uncontaminated controls (Figure 2a,b). However, growing mycoplasma in a stainless‐steel or glass bioreactor creates risks for successive reuse in other experiments. Thus, we opted to use single‐use, perfusion bioreactors to maintain process control, provide the CHO cells adequate nutrients and waste removal for optimal growth, and at the same time allow for post‐use system disposal, decomplicating lab operations.

For Day 2‐High and Day 3‐High, the early‐stage contamination event models, initial CHO cell viabilities decreased to below 3 standard deviations of the mean of the control runs (88.86%) by 3.67 and 3.21 days, respectively, after the introduction of *M. arginini* to the bioreactor (Figure 2c,d). In contrast, for bioreactors Day 9‐Low and Day 12‐High, the late‐stage contamination events, the first decrease in viability was observed after 5.71 and 5.90 days, respectively, after introduction of *M. arginini* (Figure 2e,f). Our data indicate that the conditions in the bioreactor, including but not limited to VCD, growth phase, and perfusion rate, are important factors in estimating how quickly viability will decrease after introduction of *M. arginini*. In addition, the difference in initial concentrations of *M. arginini* between Day 12‐High than Day 9‐Low, has no significance in the timing of the loss of viability.

The CHO cell growth profile after early‐stage contamination remained consistent with control bioreactors for 2 days with a 1–2‐day plateau in growth followed by decreasing for 4–5 days (Figure 2c,d). The growth profile of the CHO cells was similar during late‐stage contamination, but the viability had a more gradual decrease (Figure 2e,f). The *M. arginini* spiked CHO cell growth profile remained comparable to the control CHO cell growth profiles for 4 days. The subsequent 2‐day plateau was followed by a decrease in viability. Based on these findings, a plateau or decrease in growth profile is more indicative than viability in determining that culture conditions are deviating from established quality parameters.

**Figure 3:**
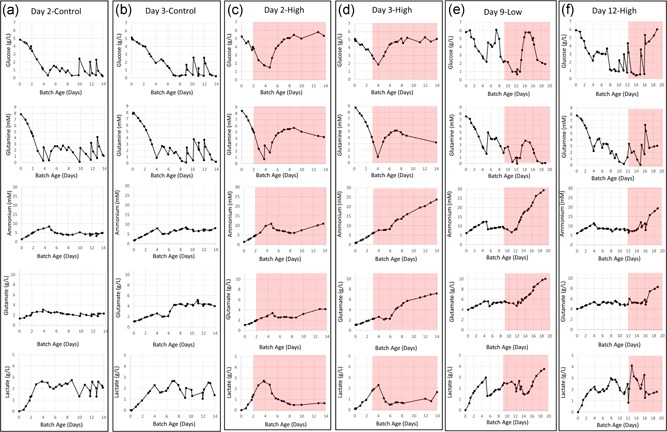
Glucose, glutamine, ammonium, glutamate, and lactate profiles for CHO cell bioreactors with and without *Mycoplasma arginini* contamination. (a) Day 2‐Control. (b) Day 3‐Control. (c) Day 2‐High. (d) Day 3‐High. (e) Day 9‐Low. (f) Day 12‐High. Red background signifies time after *M. arginini* introduction to the bioreactor. CHO, Chinese hamster ovary [Color figure can be viewed at wileyonlinelibrary.com]

Concentrations of glucose and glutamine in control bioreactors and contaminated bioreactors (Figure 3) follow the trends and timing of CHO cell growth profiles. In other words, when the CHO cell growth plateaued, glucose and glutamine concentrations began to increase, presumably because these nutrients in the perfused media were no longer being consumed as rapidly by the stationary phase CHO cells. Likewise, when the CHO cell growth plateaued, lactate concentrations decreased and leveled off as expected.

It is important to note that glucose would be rapidly consumed as a carbon source by common bacterial contaminants from skin or soil such as *Staphylococcus* sp. and *Bacillus* sp. (Baumstummler et al., 2010; Strasters & Winkler, 1963). In commercial settings, rapid drops in glucose, pH, and DO are often relied on as signs of contamination. Because *M. arginini* does not metabolize glucose (Sugimura, Ohno, Azuma, & Yamamoto, 1993), glucose exhaustion is not likely an indicator of an *M. arginini* contamination. Glucose in the bioreactors rose to 5–6 g/L approximately 4–5 days after contamination of all cultures studied (Figure 3), indicating little or no net consumption of glucose in the cultures as the overall metabolism in the culture shifts from glucose‐consuming CHO cells to non‐glucose consuming *M. arginini*.

In comparison, glutamine only rose to a steady‐state of approximately half the concentration of the perfusion media, indicating that the mycoplasma were consuming glutamine as an energy source and producing ammonia and glutamate as end products (Figure 3). This is expected given well‐understood metabolic pathways (Smith, 1955, 1957a, 1957b).

**Figure 4:**
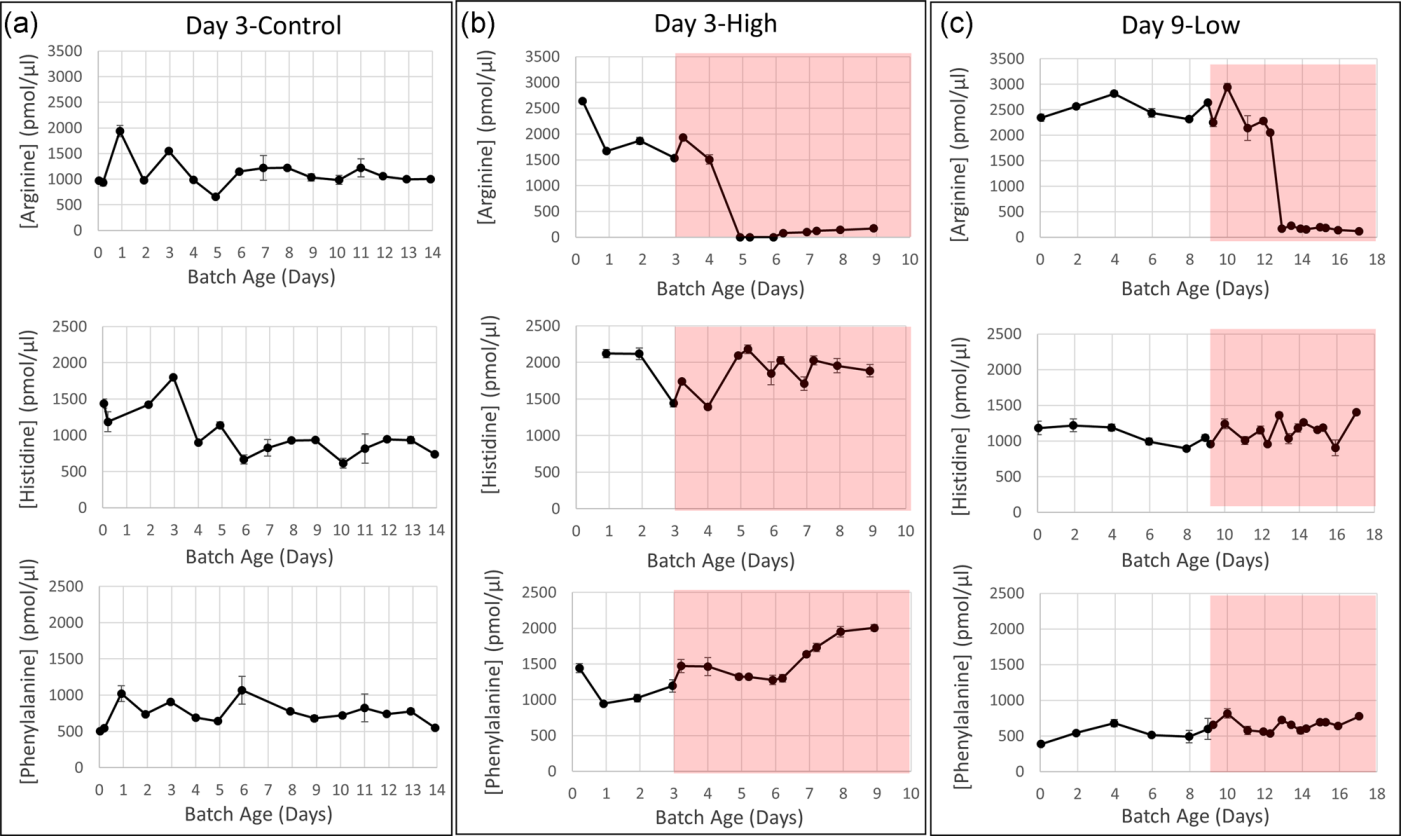
Arginine, histidine, and phenylalanine profiles for CHO cell bioreactors with and without *Mycoplasma arginini* contamination. Arginine concentrations drop by >90% as *M. arginini* reaches high titers in contaminated cultures, while essential amino acids, histidine and phenylalanine shown here as examples, remain consistent with pre‐contamination levels. (a) Day 3‐Control. (b) Day 3‐High. (c) Day 9‐Low. Red background signifies time after *M. arginini* introduction to the bioreactor. CHO, Chinese hamster ovary [Color figure can be viewed at wileyonlinelibrary.com]

The metabolites most likely to be useful as sentinels for mycoplasma contamination were ammonia which, in general, steadily increased in the culture once *M. arginini* was present (Figure 3c–f), and arginine, which rapidly declined by >90% 3–4 days into *M. arginini* presence (Figure 4b,c). Of the 15 amino acids we were able to accurately measure within the in‐process samples, only arginine decreased drastically after *M. arginini* was spiked into the bioreactors. The main pathways for ammonia are arginine and glutamine metabolism. Arginine degradation is a significant metabolic process for most mycoplasma species and is carried out through a set of three reactions that ultimately result in production of ornithine, ATP, ammonia, and carbon dioxide (Schimke & Barile, 1963). The high concentrations of ammonia by‐product in the cultures, reaching 25–30 mM despite perfusion rates of up to 3.5 CV/day, could alone be the cause for CHO cell death in the bioreactors, as ammonia has been shown to have negative effects on other mammalian cells (hybridomas) in the range of 2–10 mM (Ozturk, Riley, & Palsson, 1992). CHO cells are susceptible at concentrations as low as 4 mM (Kurano, Leist, Messi, Kurano, & Fiechter, 1990). Ammonia production by one species of mycoplasma, *M. salivarium*, has been postulated to be a virulence mechanism in patients with oral infections (Matsuura, Seto, & Watanabe, 1990). It is also important to note that ammonia and glutamate concentrations in the prepared starting media and perfused media were higher in Bioreactors Day 9‐Low and Day 12‐High than in Bioreactors Day 2‐High, Day 2‐Control, Day 3‐High, and Day 3‐Control (Figure 3). This was confirmed to be due to a change in soy hydrolysate source, which appeared to result in both a slower entrance into exponential growth as well as a decrease in exponential growth rate before spiking.

### 3.3 Effects of *M. arginini* on culture conditions and process controls

**Figure 5:**
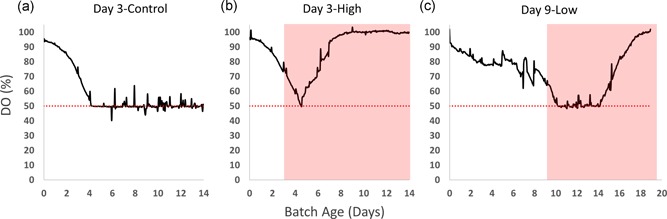
DO profiles for CHO cell bioreactors with and without *Mycoplasma arginini* contamination. (a) Day 3‐Control, (b) Day 3‐High, and (c) Day 9‐Low. Red background signifies time after *M. arginini* introduction to the bioreactor. The red dotted line indicates the 50% DO setpoint. CHO, Chinese hamster ovary; DO, dissolved oxygen [Color figure can be viewed at wileyonlinelibrary.com]

**Figure 6:**
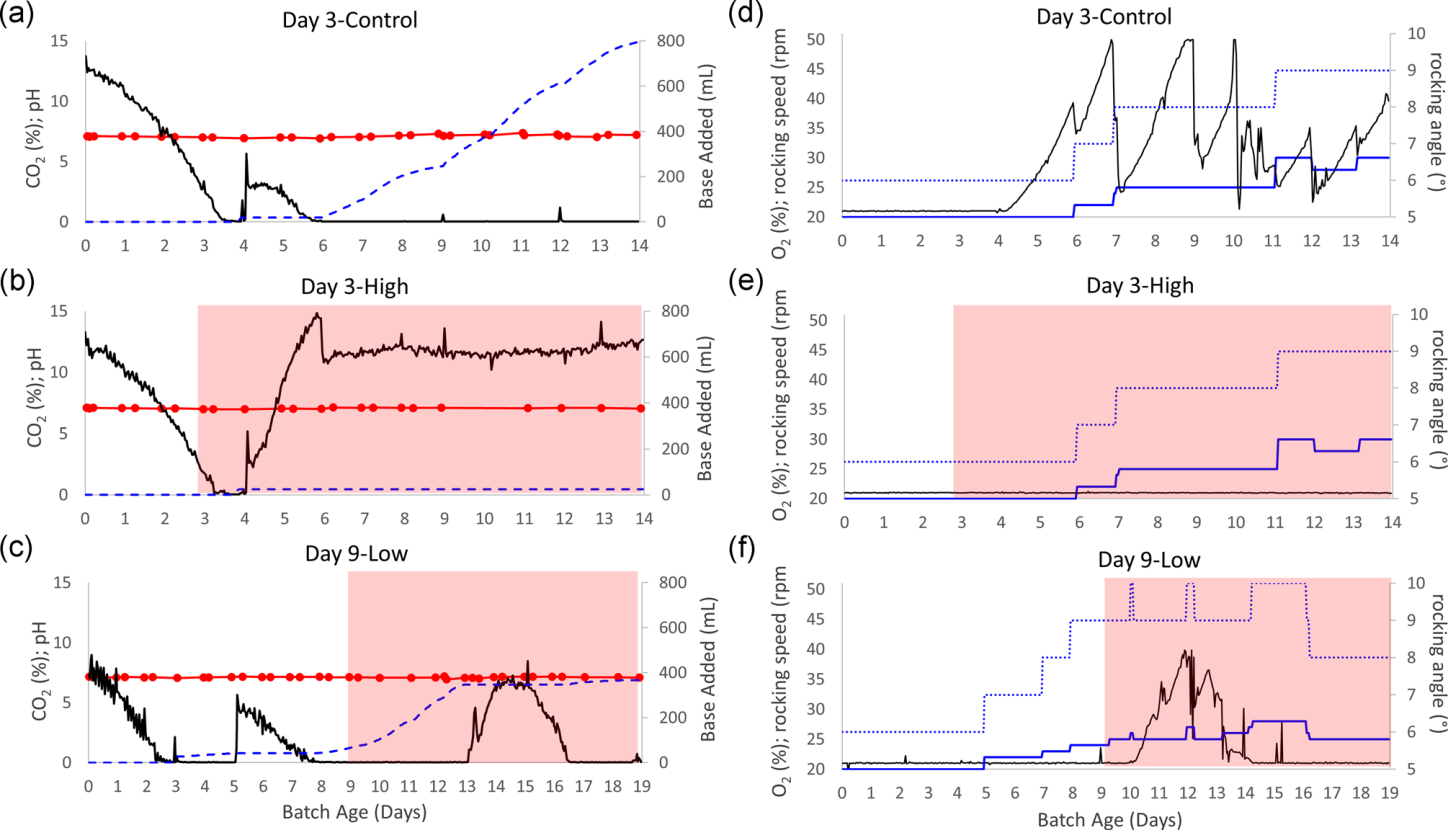
pH and DO control profiles for CHO cell bioreactors with and without *Mycoplasma arginini* contamination. (a, d) Day 3‐Control, (b, e) Day 3‐High, and (c, f) Day 9‐Low. pH (red) control was maintained through CO_2_ gassing (black) and addition of 0.5 M NaOH (blue dash). DO was maintained through O_2_ gassing (black) and manual control of rocking speed (blue solid line) and rocking angle (blue dotted line). Red background signifies time after *M. arginini* introduction to the bioreactor. CHO, Chinese hamster ovary; DO, dissolved oxygen [Color figure can be viewed at wileyonlinelibrary.com]

In commercial settings, critical process parameters are consistently collected in real‐time with automatic feedback to control the process. Thus, we closely monitored any changes in pH and dissolved oxygen (DO) after *M. arginini* contamination to investigate if they could serve as sentinels for contamination. The DO setpoint was 50% of air saturation and was controlled automatically with O_2_ overlay, and manually with rocking speed and rocking angle (Table 1). In control bioreactors, DO gradually equilibrates to the 50% setpoint over 4–5 days of culture and remains at or above 50% for the duration of the runs (Figure 5a). To maintain 50% air saturation, the control system automatically adds O_2_ into the gas mix to increase DO. When possible, the rocking speed and/or angle can be manually increased when O_2_% reaches 30–40% because if the O_2_% reaches its maximum of 50%, the system loses its ability to control for increasing oxygen demand (Figure 6d–f). In early‐stage contamination event models, DO began increasing after just less than 2 days after the *M. arginini* spike (Figure 5b), indicating little oxygen consumption by the culture. In late‐stage contamination event models, DO began to increase 4–5 days after *M. arginini* spike (Figure 5c). Overall, we found that timing of the DO accumulation was directly related to the culture conditions, namely CHO VCD and perfusion rate, as the *M. arginini* took over the bioreactor culture and overall oxygen consumption ceased.

In control bioreactors, pH control via CO_2_ overlay and 0.5 M NaOH addition followed similar trends. In the first few culture days, CO_2_ overlay was sufficient to maintain pH of 7.1. After the cells reached approximately 10 million cells/ml, base additions were required and continued for the remainder of the run (Figure 6a). In early stage contamination event models, CO_2_ percentage began to rise 1–2 days after mycoplasma was spiked into the bioreactor, which corresponded to the approximate time when the CHO cell growth rate slowed, and the addition of base was never required (Figure 6b). In late‐stage contamination event models, this process lagged as CO_2_ increased 4 days after the cultures were spiked with mycoplasma and base additions were still required (Figure 6c).

### 3.4 CHO cell IgG1 production after contamination with *M. arginini*

**Figure 7:**
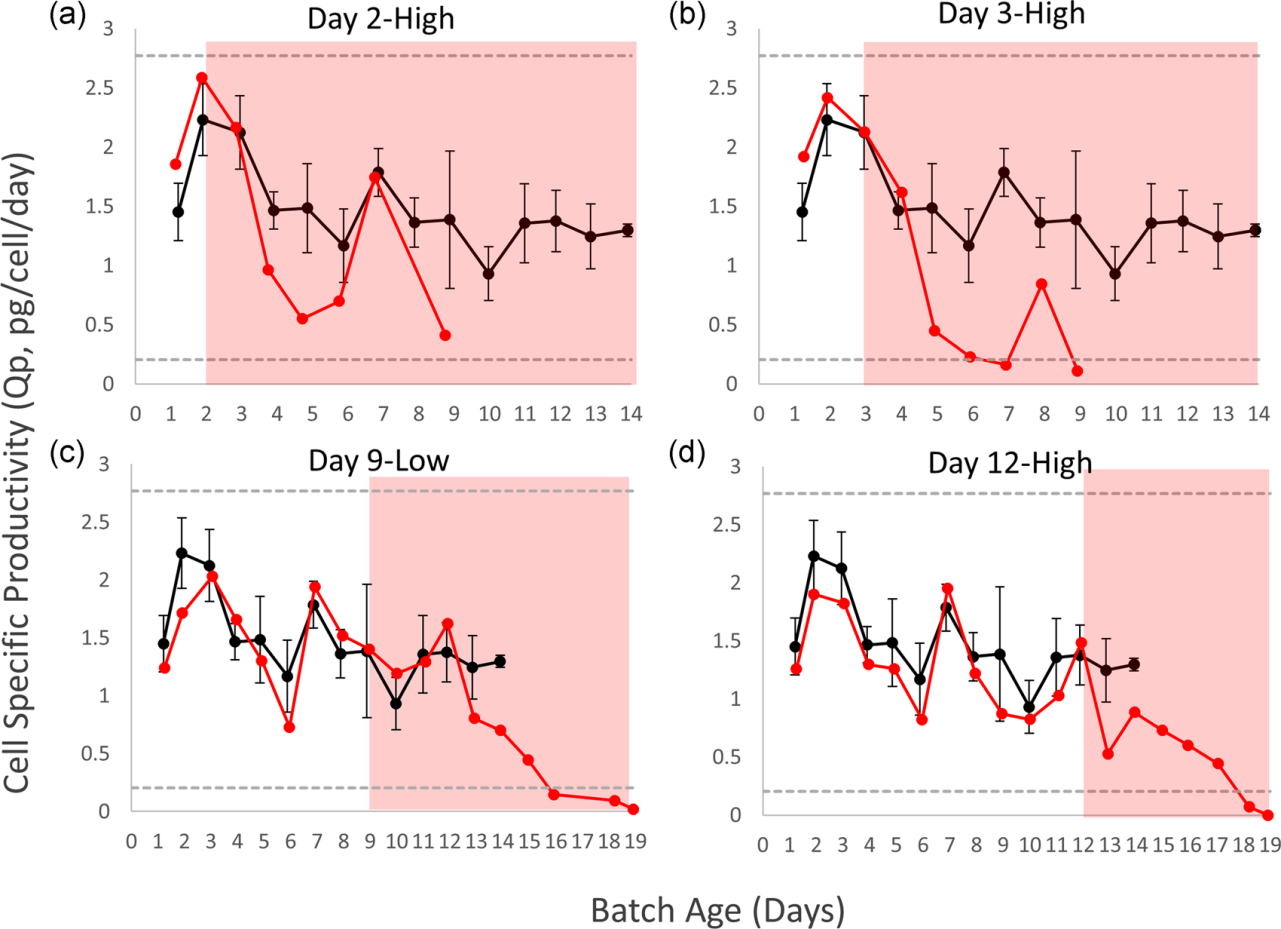
Cell‐specific productivity profiles for CHO cell bioreactors with and without *Mycoplasma arginini* contamination. The average *Q*_p_ of the control bioreactors over batch age is shown in each graph in black with error bars indicating ± 1 standard deviation of the mean. The grey dashed lines indicate ± 3 standard deviation of the mean of the average of the *Q*_p_ of the control bioreactor runs. *Q*_p_ of CHO cells in *M. arginini*‐contaminated bioreactors are shown in each graph in red. (a) Day 2‐High. (b) Day 3‐High. (c) Day 9‐Low. (d) Day 12‐High. Red background signifies time after *M. arginini* introduction to the bioreactor. CHO, Chinese hamster ovary [Color figure can be viewed at wileyonlinelibrary.com]

IgG1 production from the uncontaminated CHO DG44 cell averaged 1.49 ± 0.41 pg·cell^−1^·day^−1^, with variability likely influenced by multiple factors including cell density, growth rate, and perfusion rate. This variability made it challenging to determine if *M. arginini* contamination specifically caused any changes to productivity. However, based on the comparison of parameters and measuring *Q*_p_ after *M. arginini* contamination relative to uninfected controls, the CHO cells maintained some productivity even as the health of the culture declined (Figure 7).

In the early‐stage contamination model, the CHO cell density was as low as 3.7 × 10^5^ cells/ml and the reduced IgG concentration resulted in a less precise *Q*_p_ calculation. However, it was evident that the viable cells maintained a close to average productivity for some duration after the *M. arginini* spike. In Day 2‐High, *Q*_p_ of the spiked culture was significantly lower 2 days after spiking but appears to have recovered 5 days after the spike. One week after the spike on Day 9, the *Q*_p_ of the spiked culture was significantly lower than the uncontaminated control because after Day 9 there were too few viable CHO cells left in the bioreactor to make accurate calculations (less than 100,000 CHO cells) (Figure 7a). In Day 3‐High, *Q*_p_ between the spiked and uninfected cultures were significantly different. The *Q*_p_ of the spiked culture dropped below 3 standard deviations to roughly 15% of the average *Q*_p_ of the uninfected cultures 4 days after contamination (Figure 7b). Day 3‐High had the most significant decrease in *Q*_p_ of all bioreactors, which may have been caused by the proximity in timing of the mycoplasma contamination event and the start of perfusion.

In the late‐stage contamination event models, the higher CHO cell densities and higher volumetric titer resulted in more precise *Q*_p_ measurements. In Day 9‐Low, *Q*_p_ decreased from 3 to 6 days after contamination, but remained within 3 standard deviations of the average of the control runs until 7 days after *M. arginini* contamination when *Q*_p_ dropped to 10% of the average of the uninfected cultures (Figure 7c). In Day 12‐High, the *Q*_p_ of the infected culture was slightly below the average after mycoplasma contamination but was not significantly lower until 6 days after spiking with *M. arginini* (Figure 7d). Overall, *Q*_p_ does not appear to be a suitable indicator for early presence of *M. arginini*. Because IgG product is still produced in *M. arginini*‐infected cultures, vigilance is warranted when screening harvests in commercial settings as impacted product could otherwise be further processed downstream into final product.

## 4 CONCLUSION

One mycoplasma species, *M. arginini*, grew and persisted in a serum‐free bioreactor culture of a CHO DG44 cell line expressing a model monoclonal IgG1 antibody. We investigated the effects of mycoplasma presence on CHO cell health, productivity, culture metabolism, and process parameters compared to uninfected controls and found that effects are seen 2–6 days after *M. arginini* introduction to the culture. CHO cell growth, which is dependent on the VCD and perfusion conditions at the time of contamination, plateaued 2–4 days after contamination, and viability began to decline after 3–6 days. As CHO cell growth rates declined, so did the consumption of glucose and glutamine. Additionally, decreased growth rates and eventual decline of the CHO cells led to changes in process conditions and corresponding controls, such as DO and pH, which were indicators of contamination as early as 2 days after *M. arginini* introduction. Growth of *M. arginini* resulted in consumption of high concentrations of arginine and surely contributed to a very high accumulation of ammonia, which in turn creates a toxic environment for CHO cells. Of major importance, there were no changes seen in CHO cell health or process conditions until at least 2 days after the introduction of *M. arginini* to the bioreactor, and for other mycoplasma species, it is possible that CHO cell growth may not be affected. This 2‐day period, or any period before change in CHO cell growth, should continue to be of critical interest for early detection of a mycoplasma contamination event, in lieu of validated rapid microbial methods. Although this study was conducted using a single mycoplasma species, this provides an understanding for earlier detection of mycoplasma contamination in biomanufacturing and indicates how real‐time process monitoring can identify a mycoplasma contamination event in the manufacturing environment.

## CONFLICT OF INTERESTS

The authors declare that there is no conflict of interests.

## DISCLAIMER

This publication reflects the views of the author and should not be construed to represent FDA's views or policies.
